# Multi-scale analysis reveals key targets mediating BPA-induced sensorineural hearing loss

**DOI:** 10.1097/MD.0000000000049132

**Published:** 2026-06-05

**Authors:** Xin Yan, Junmei Xuan, Jianghua Peng

**Affiliations:** a Department of Otolaryngology, Shaoxing People’s Hospital (The First Affiliated Hospital, Shaoxing University), Shaoxing, China; b Department of General Practice, Shaoxing People’s Hospital (The First Affiliated Hospital, Shaoxing University), Shaoxing, China.

**Keywords:** bisphenol A, dynamic simulation, genetic susceptibility, Mendelian randomization, molecule, sensorineural hearing loss, toxicity

## Abstract

The mechanism of bisphenol A (BPA) on sensorineural hearing loss (SNHL) remains undefined. This study investigates BPA’s toxic mechanism on SNHL. ProTox database was performed to analyze the toxicity of BPA. Intersection genes were screened using network toxicology, and causal genes associated with SNHL were identified through Mendelian randomization. The ligand–protein binding activity was validated through molecular docking and dynamic simulation. BPA showed a toxicity classification of Class 4, with toxicological profiles involving the blood–brain barrier, mitochondrial membrane potential, and estrogen receptor alpha. A total of 92 BPA target genes were found to be related to SNHL. These were enriched in potassium ion channel processes and MAPK, PI3K–Akt pathways. Two-sample Mendelian randomization identified 3 causal genes, with small effect sizes: MANBA (odds ratios [OR] = 0.950, *P* = .009), PDE6D (OR = 1.055, *P* = .001), vascular endothelial growth factor A (OR = 1.030, *P* = .022). Molecular docking with BPA revealed minimum binding free energies of ‐8.1, ‐6.7, and ‐6.1 kcal/mol; MANBA–BPA binding was stable in dynamics simulations. BPA can exert toxic effects on SNHL through potassium channel related processes, as well as MAPK and PI3K–Akt signaling pathways. MANBA, PDE6D, and vascular endothelial growth factor A also play key mediating roles in this process.

## 1. Introduction

Hearing loss affects approximately 6.8% of the global population and ranks as the fourth leading cause of years lived with disability worldwide. With a higher prevalence in males and older adults, sensorineural hearing loss (SNHL) accounts for over 90% of all hearing loss cases, underscoring its dominant role in the global burden of hearing impairment.^[1]^ As the main type of hearing loss, SNHL not only leads to speech communication disorders and social dysfunction, but also significantly correlates with cognitive decline and increased risk of dementia in untreated cases.^[2]^ The pathogenic mechanism of SNHL includes the following factors: genetic factors such as GJB2 gene mutations can cause abnormal development of the inner ear.^[3]^ The degenerative changes of inner ear hair cells during aging are an important pathological basis.^[4]^ Among environmental factors, long-term noise exposure^[5]^ and exposure to ototoxic drugs (such as cisplatin)^[6]^ can directly lead to apoptosis of cochlear hair cells and damage to spiral ganglion neurons.^[7]^ Notably, SNHL is a multifactorial progressive disorder resulting from the complex interplay of genetic and environmental factors, with oxidative stress and mitochondrial damage recognized as key pathological mechanisms underlying its onset and progression.^[8]^

In the process of industrialization, environmental pollutants such as organophosphate insecticides, bisphenol A (BPA), Heavy metals are widely spread through air, water, and the food chain, and environmental exposure has been proven to be one of the important pathogenic factors at risk of hearing loss.^[9]^ Ototraumatic chemicals like BPA, as pervasive environmental pollutants, can induce auditory dysfunction through mechanisms including oxidative stress and ion channel disruption.^[10]^ BPA, as a widely used industrial compound, is mainly employed for manufacturing polycarbonate plastics and epoxy resins. Its derivatives are commonly found in daily necessities such as food packaging and drinking water pipelines.^[11]^ Environmental exposure studies have shown that BPA can enter the food chain through plastic degradation, additive leakage, and other pathways, leading to sustained low-dose exposure in the population.^[12]^ In recent years, research has suggested a correlation between BPA exposure and hearing loss: a cross-sectional study of children found a negative correlation between urinary BPA concentration and cochlear outer hair cell activity, especially in the moderate frequency hearing range, and this effect may be mediated through the oxidative stress pathway.^[13]^ NHANES data analysis also showed a trend association between BPA and adult high-frequency hearing loss, but existing evidence is mostly limited to epidemiological relevance and lacks in-depth exploration of causal mechanisms.^[14]^ At the level of animal experiments, a study on larval zebrafish^[15]^ have found that exposure to BPA can induce dose- and time-dependent loss of lateral line hair cells and suppress the regeneration of damaged hair cells, with oxidative stress identified as the key mediating pathway. This result suggests that BPA may exert direct toxic effects on auditory sensory cells and impair their repair capacity in vertebrates, including mammals. Although it cannot be directly extrapolated to humans at present, it also provides a new mechanistic direction for the study of BPA-induced sensorineural hearing loss. A recent in vitro study further validated that BPA directly targets cochlear hair cells (HEI-OC1), where it activates estrogen receptor α (ER-α) and triggers mitochondrial reactive oxygen species production, ultimately inducing pyroptosis.^[16]^ There are also studies suggesting that BPA, as an endocrine disruptor, may disrupt the balance of hormones in the human body, potentially affecting health. However, the specific endocrine disruption mechanism of BPA in hearing loss is still unclear.^[17]^ F á belov á et al pointed out that BPA may indirectly affect the development and function of the cochlea by interfering with thyroid hormone homeostasis, activating aromatic hydrocarbon receptors, or inducing oxidative stress,^[18]^ but the specific molecular pathways and key gene mechanisms have not been thoroughly explored.

Existing research^[9,13,18]^ has confirmed a significant association between BPA as an environmental ototoxic substance and SNHL, but it is mostly limited to exposure effect association analysis, lacking an explanation of the mechanism by which BPA affects the auditory system through gene regulation, especially in key gaps in target gene screening, cause inference, and molecular action pathways. This study integrates network pharmacology, Mendelian randomization (MR), molecular docking, and dynamic simulation methods to systematically explore the molecular mechanism of BPA induced SNHL from the perspective of gene environment interaction for the first time. The aim is to fill the theoretical gap in the analysis of pathogenic pathways in existing research and provide innovative ideas for the precise mechanism study of ototoxicity of environmental pollutants.

## 2. Methods

To clarify the pathogenic mechanism of BPA-induced SNHL, this study integrates multiple analytical approaches following a multi-scale progressive logic: environmental exposure triggers molecular perturbations, which interact with genetic susceptibility to drive cellular dysfunction. Specifically, we first identified BPA’s molecular toxicological targets via toxicity prediction; then screened BPA–SNHL common target genes using network toxicology to reveal core linking pathways; verified causal gene–SNHL associations by MR to define genetic susceptibility roles; and finally validated key gene-BPA binding stability through molecular docking and dynamic simulation. These scales are hierarchically connected, with molecular pathways as the core bridge between environmental exposure and cellular damage.

### 2.1. Network toxicology analysis

*Toxicological analysis*: we searched for the SMILE structure and 3D molecular structure of BPA using the PubChem database, and conducted toxicity analysis of BPA via the ProTox database.

*Screening of genes related to BPA targets and SNHL*: we screened genes related to SNHL using the OMIM database, merged genes related to predicting SNHL with a GeneCards relevance score >10 (a widely adopted threshold to filter high-confidence disease–gene associations by excluding low-specificity, spurious gene matches with weak biological relevance),^[19]^ predicted BPA target degeneracy via the Chembl, STITCH, and SWISS databases, and intersected BPA target genes with disease-related genes to obtain overlapping genes.

*Gene ontology (GO) enrichment analysis*: GO enrichment analysis was performed on the obtained intersection. Firstly, we converted the intersection gene ID to GO ID using the reference database org.Hs.e.g..db (Human Species Annotation Package). Then, the multiple hypothesis testing using BH correction was performed to control for false discovery rate (FDR); *Q*value < 0.05 was used as the threshold for significant enrichment, and GO entries with statistical significance were selected. The enrichment results were presented in 3 categories: biological process, cellular component, and molecular function.

*Kyoto Encyclopedia of Genes and Genomes (KEGG) enrichment analysis*: ensembl ID was converted into the gene ID format required by KEGG, using the reference database org.Hs.e.g..db (Human Species Annotation Package). BH correction conducted multiple hypothesis testing to control for FDR; *Q*value < 0.05 was used as a screening condition for significant enrichment. Enrichment results from KEGG pathway database. Identify significantly enriched pathways through screening.

### 2.2. Mendelian randomization

*Data acquisition*: the gene expression quantitative trait locus (eQTL) data was sourced from the eQTLGen database, with the sample size of 31,684. The GWAS data for SNHL was sourced from the FinnGen database, with the sample size of 482,076. Both sets of data were sourced from the European population.

*Two sample MR analysis*: the cis-eQTL data of genes in the eQTLGen database were searched for as exposure (extracting 100 kb upstream and downstream gene range) from the intersection genes mentioned above. To avoid linkage imbalance, the threshold for single nucleotide polymorphism (SNP) to perform club was set to kb = 100, *r*^2^ = 0.3, *P* < 5e‐8, and the *F*-statistic value was set to >10 to prevent the occurrence of bias induced by weak instrumental variables (IVs). The GWAS data of SNHL was used as the outcome MR Egger, weighted median, inverse variance weighted (IVW), simple mode, and weighted mode. If the IVW method had a *P* <.05 and the 5 methods had the same direction of action, it was preliminarily believed that the exposure had a causal effect on the outcome.

*Sensitivity analysis*: we used the MR Egger intercept method and MR-Presso global test to exclude horizontal pleiotropy, Cochrane *Q* test to exclude heterogeneity, and both evaluated the robustness of the results with a *P*-value >.05. The “leave-one-out” sensitivity analysis was used to check for biased SNPs.

Based on the fulfillment of both primary analysis and sensitivity assessment criteria, a conclusive causal relationship between exposure and outcome was established.

Enrichment analysis and MR were performed using R4.4.2 and related packages (such as clusterProfiler, clusterProfiler, and TwoSampleMR).

### 2.3. Molecular docking

Genes were selected based on MR from the RCSB Protein Data Bank (PDB) database Or AlphaFold database. We searched for the molecular structure of the protein and performed blind docking with the 3D molecular structure of BPA using the CB-DOCK2 server. The binding was considered stable with a minimum binding free <‐5 kcal/mol.

### 2.4. Molecular dynamics simulation

*Protein preparation and docking*: the protein with the lowest binding free energy from molecular docking was selected. PDB files were processed using Pymol software, and atoms were added to the protein files via SPDBV-4.10. The Amber14sb force field was applied to obtain protein topology files, while ligand topology files were generated using Sobtop_1.0 software. The ligand and protein topology files were then merged.

*System setup and equilibration*: after adding a periodic box, water models, and ions, nuclear equilibration was performed. Energy minimization was conducted in 2 stages: first using the steepest descent method, followed by the conjugate gradient method. Subsequently, the system underwent pre-equilibration under NVT (constant number of particles, volume, and temperature) and NPT (constant number of particles, pressure, and temperature) ensembles.

*Molecular dynamics simulation and analysis*: molecular dynamics simulations were executed using GROMACS. Post-simulation analyses included calculations of root mean square deviation, root mean square fluctuation, protein gyration radius, and solvent-accessible surface area. A free energy landscape diagram was constructed using Python 3.9.7.

## 3. Results

### 3.1. Network toxicology analysis

**Figure 1.**
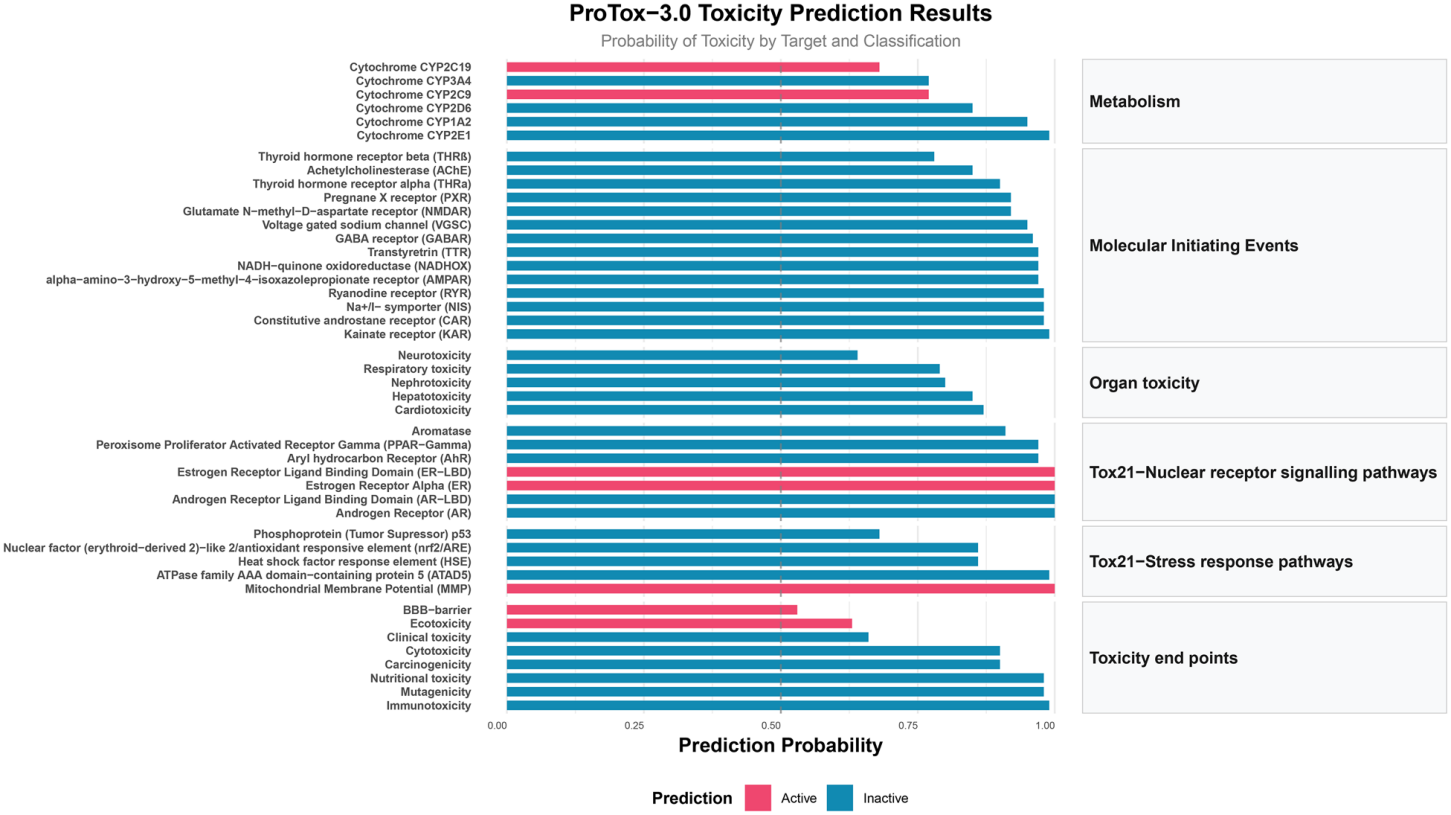
Toxicity prediction results of bisphenol A.

*Toxicity prediction*: according to the analysis results of BPA on ProTox server, its toxicity level was level 4, and the toxic targets covered multiple key biological systems and molecular targets, including: blood–brain barrier (BBB-barrier), ecotoxicity, ER-α, estrogen receptor ligand binding domain, mitochondrial membrane potential, cytochrome CYP2C19, and cytochrome CYP2C9 (Fig. 1).

**Figure 2.**
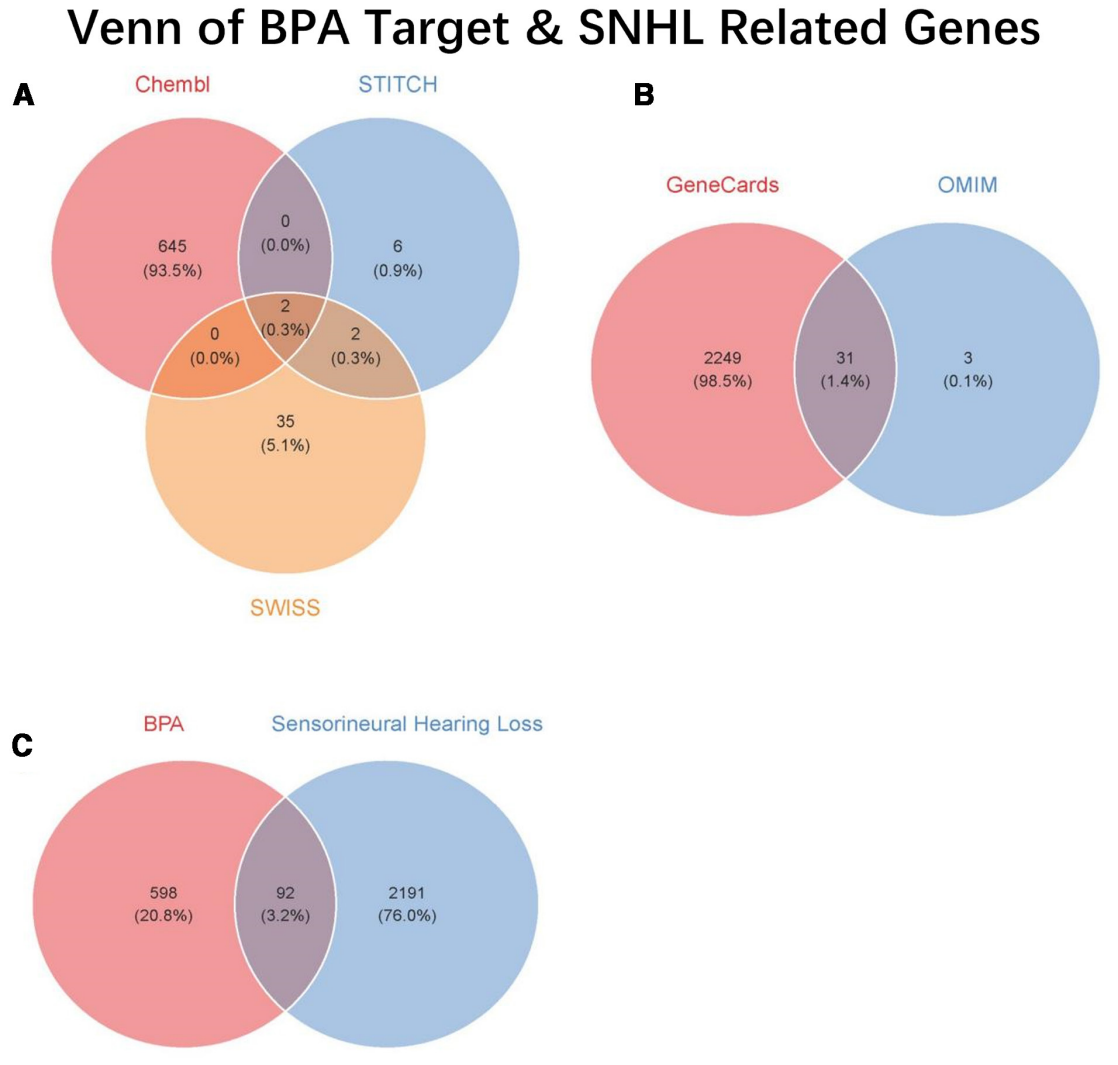
Venn diagram analysis of BPA target genes and SNHL-related genes, (A) Distribution of BPA target genes across ChEMBL, STITCH, and SWISS databases, (B) Distribution of SNHL-related genes across GeneCards and OMIM databases, (C) Overlap between BPA target genes and SNHL-related genes. BPA = bisphenol A, SNHL = sensorineural hearing loss.

*Screening of BPA targets and genes associated with SNHL*: for BPA, a total of 647 target genes were predicted by ChEMBL, 10 by STITCH, and 39 by the SWISS database. After merging the data from these 3 databases, 690 unique target genes were identified. Regarding SNHL, 2280 associated genes were retrieved from GeneCards, and 34 from OMIM. Following integration, 2283 nonredundant genes were identified. Finally, cross-referencing the BPA targets with the SNHL-associated genes revealed 92 overlapping genes (Fig. 2).

**Figure 3.**
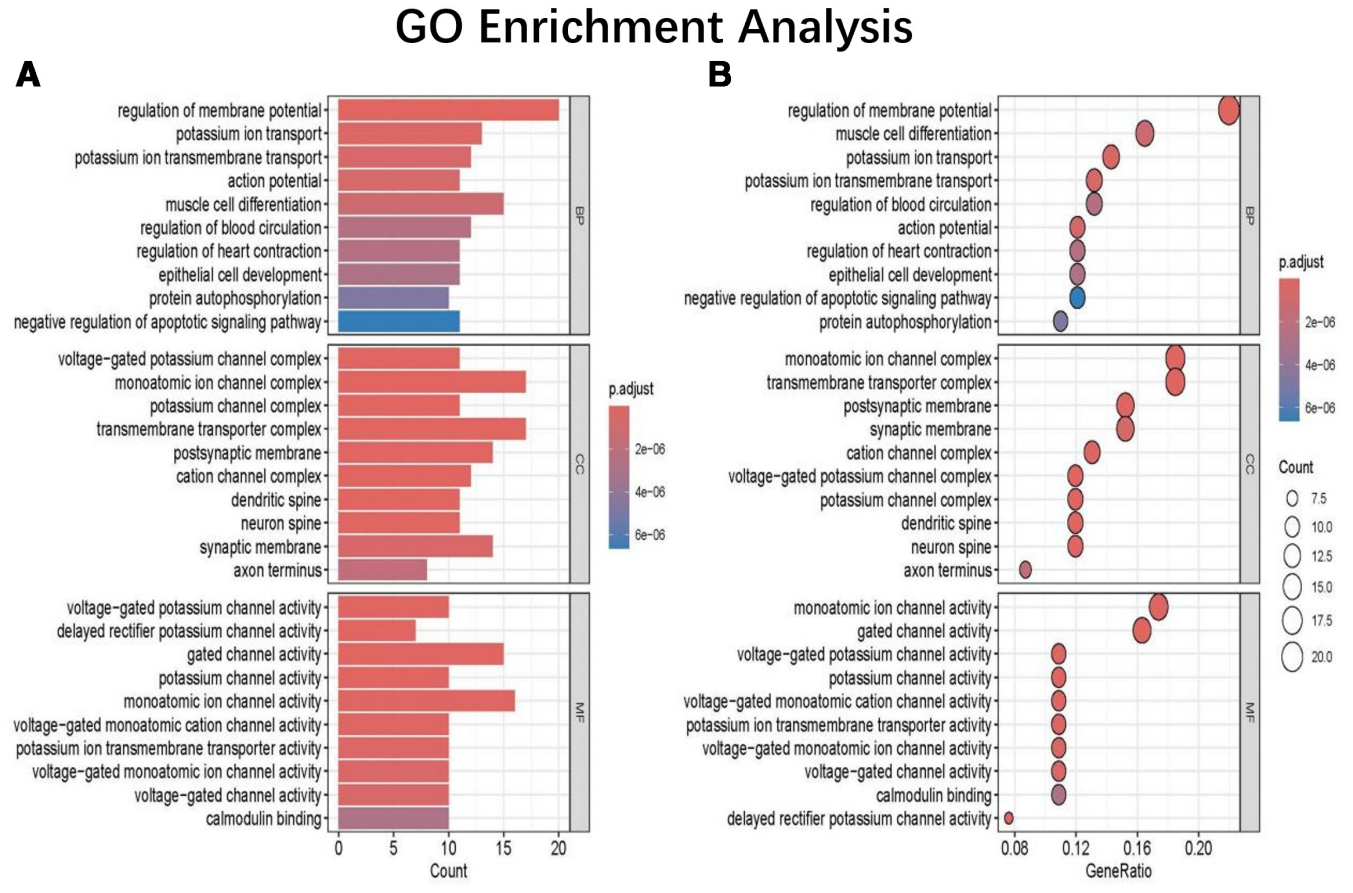
The GO enrichment analysis results, (A) barplot of GO enrichment terms with gene counts and (B) bubble plot integrating gene counts and generatio. BP = biological process, CC = cellular component, GO = gene ontology, MF = molecular function, p.adjust = adjusted *P*-value.

*GO enrichment analysis*: The main enrichment of genes occurred in primarily enriched in the biological process categories of regulation of membrane potential, potassium ion transport, potassium ion transmembrane transport. In cellular component analysis, genes were enriched in subcellular structures including synapse-related components (such as postsynaptic membrane, dendritic spine, and axon terminus), ion channel complexes (such as voltage-gated potassium channel complex and potassium channel complex), and transmembrane transporter complex. Molecular function analysis indicated that genes were mainly enriched in voltage-gated potassium channel activity and potassium ion transmembrane transporter activity (Fig. 3).

**Figure 4.**
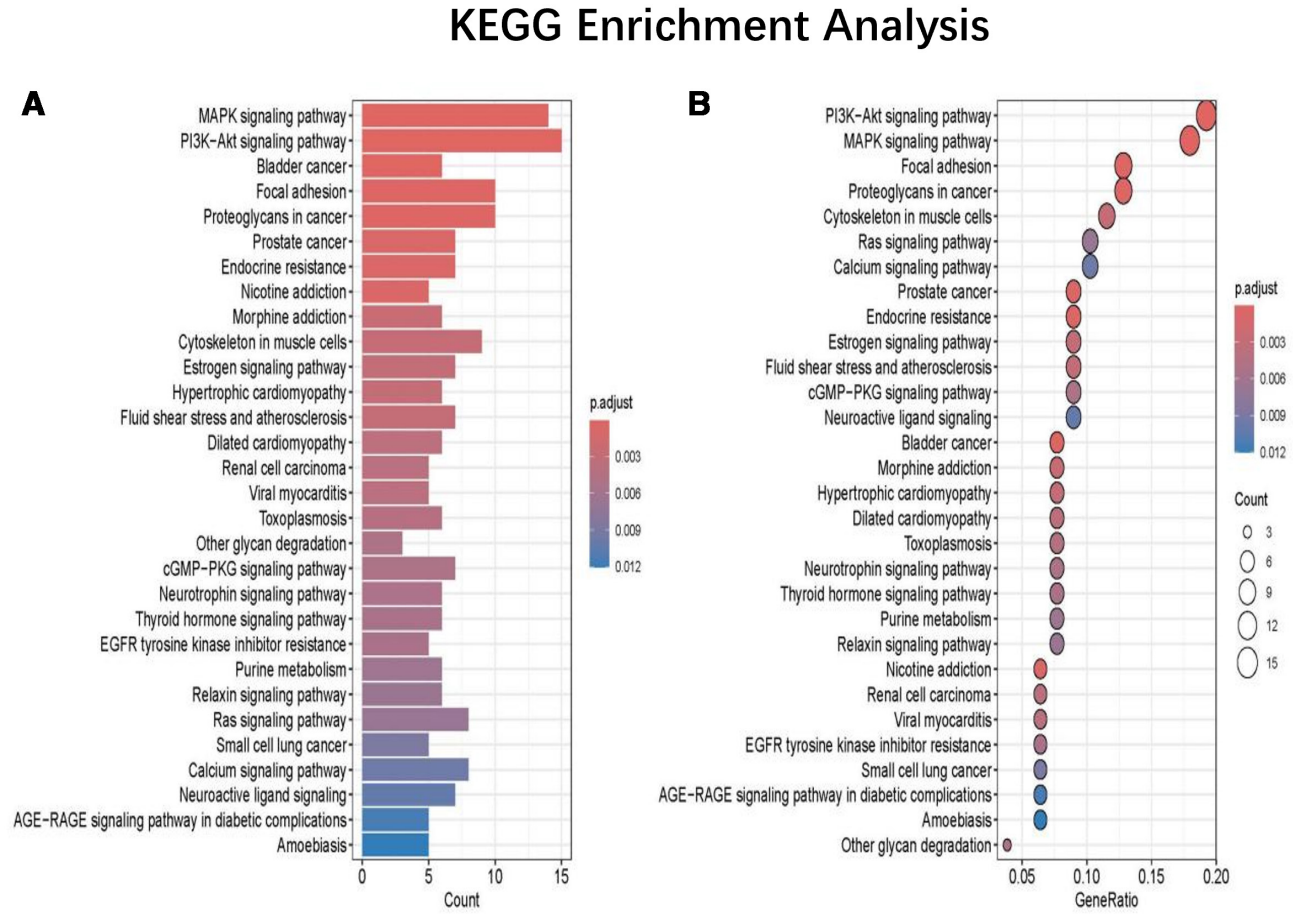
The KEGG enrichment analysis results, (A) barplot of KEGG enrichment terms with gene counts and (B) bubble plot integrating gene counts and generation. KEGG = Kyoto Encyclopedia of Genes and Genomes, p.adjust = adjusted *P*-value.

*KEGG enrichment analysis*: Genes were primarily enriched in the MAPK signaling pathway, PI3K–Akt signaling pathway, etc. (Fig. 4).

### 3.2. MR analysis

**Figure 5.**
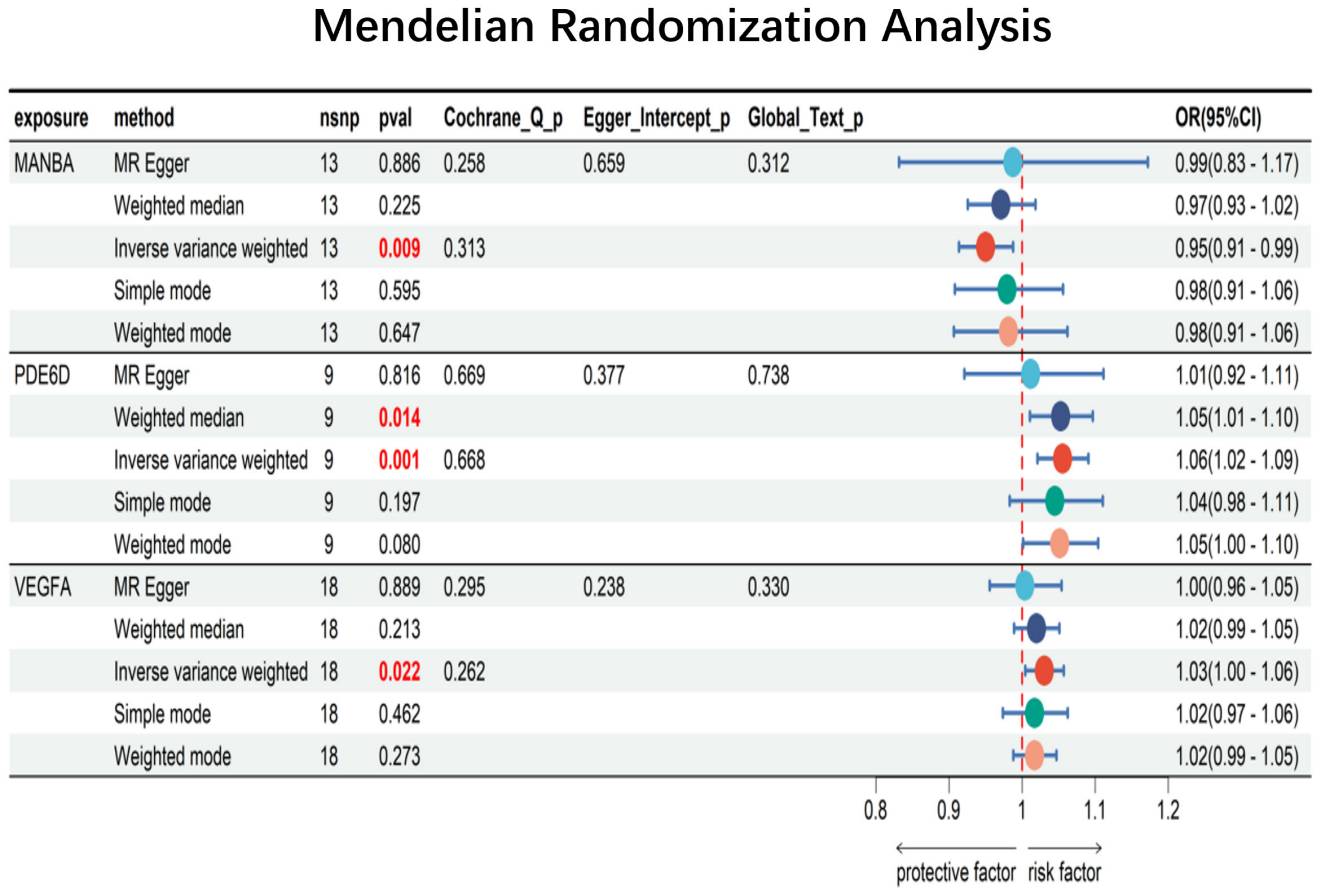
Forest plot of Mendelian randomization analysis results. CI = confidence interval, Cochrane_Q_p = the *P*-value of Cochrane *Q* test, Egger_Intercept_p = *P*-value of MR Egger intercept test, Global_Text_p = the *P*-value of the global test in MR-PRESSO analysis, MR = Mendelian randomization, nsnp = number of single nucleotide polymorphisms, OR = odds ratio, pval = value of *P*.

**Figure 6.**
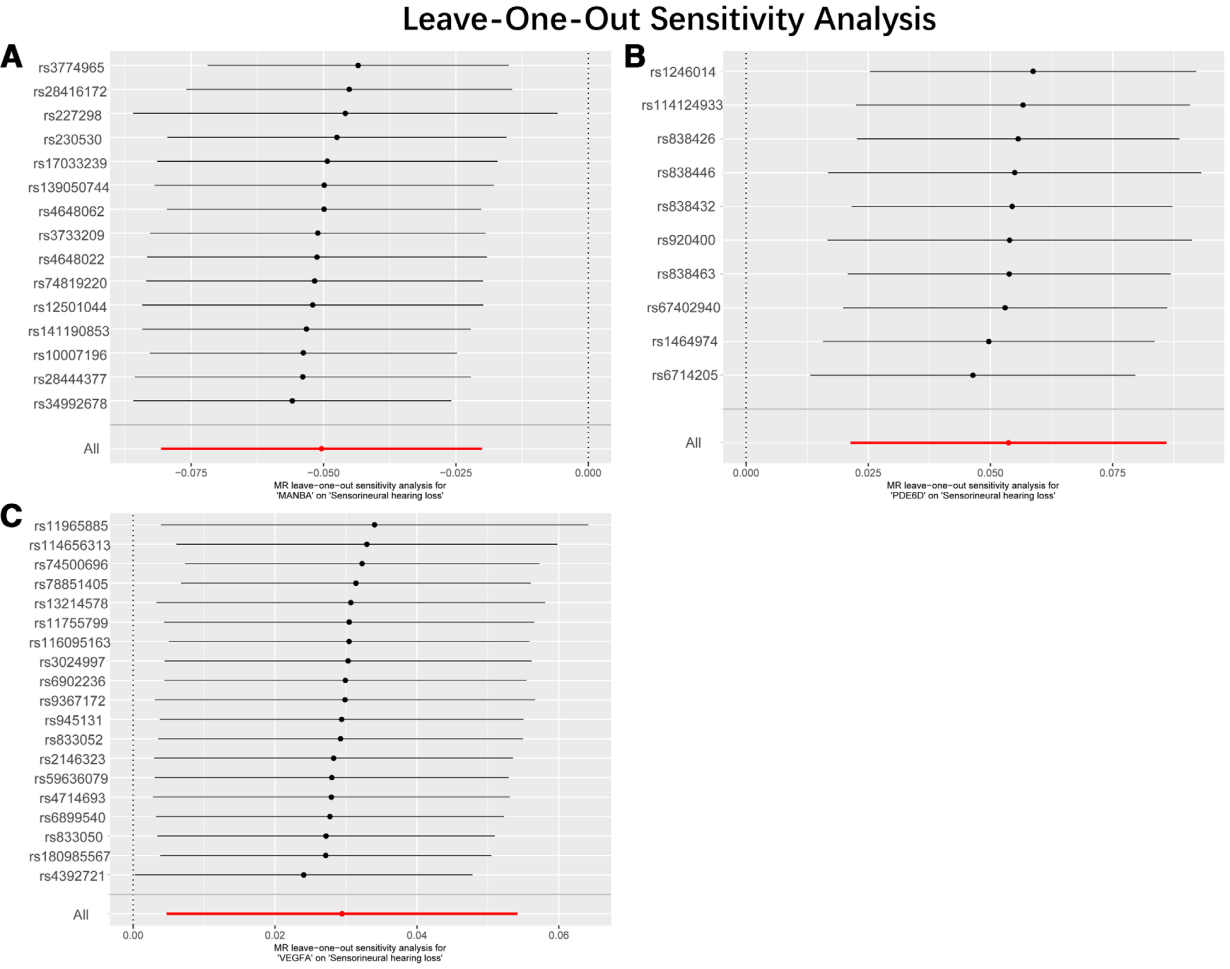
Mendelian randomization leave-one-out sensitivity analysis. (A) “MANBA” in “Sensorineural hearing loss”; (B) “PDE6D” on “Sensorineural hearing loss”; (C) “VEGFA” on “Sensorineural hearing loss.” The 95% CI and causal estimate when each SNP was eliminated individually are shown by the black bars and dots. The fixed-effect IVW method’s overall estimate and 95% confidence interval are shown by the red dot and bar. CI = confidence interval, IVW = inverse-variance weighted, SNP = single nucleotide polymorphism, VEGFA = vascular endothelial growth factor A.

Among the 92 intersecting genes, screening across the eQTLGen database yielded eQTL data for 61 genes, of which only 41 eQTLs provided sufficient SNPs as IVs for MR analysis. Following MR analysis, 3 eQTLs showed significant causal associations with SNHL, with gene IDs: MANBA (IVW, odds ratios [OR] = 0.950, 95% CI = 0.914–0.987, *P* = .009), PDE6D (IVW, OR = 1.055, 95% CI = 1.021–1.091, *P* = .001), and vascular endothelial growth factor A (VEGFA) (IVW, OR = 1.030, 95% CI = 1.004–1.057, *P* = .022). Pleiotropy and heterogeneity were excluded based on sensitivity analysis *P*-values (Fig. 5). Leave-one-out sensitivity analysis indicated no obvious outlier SNPs (Fig. 6).

### 3.3. Molecular docking

**Figure 7.**
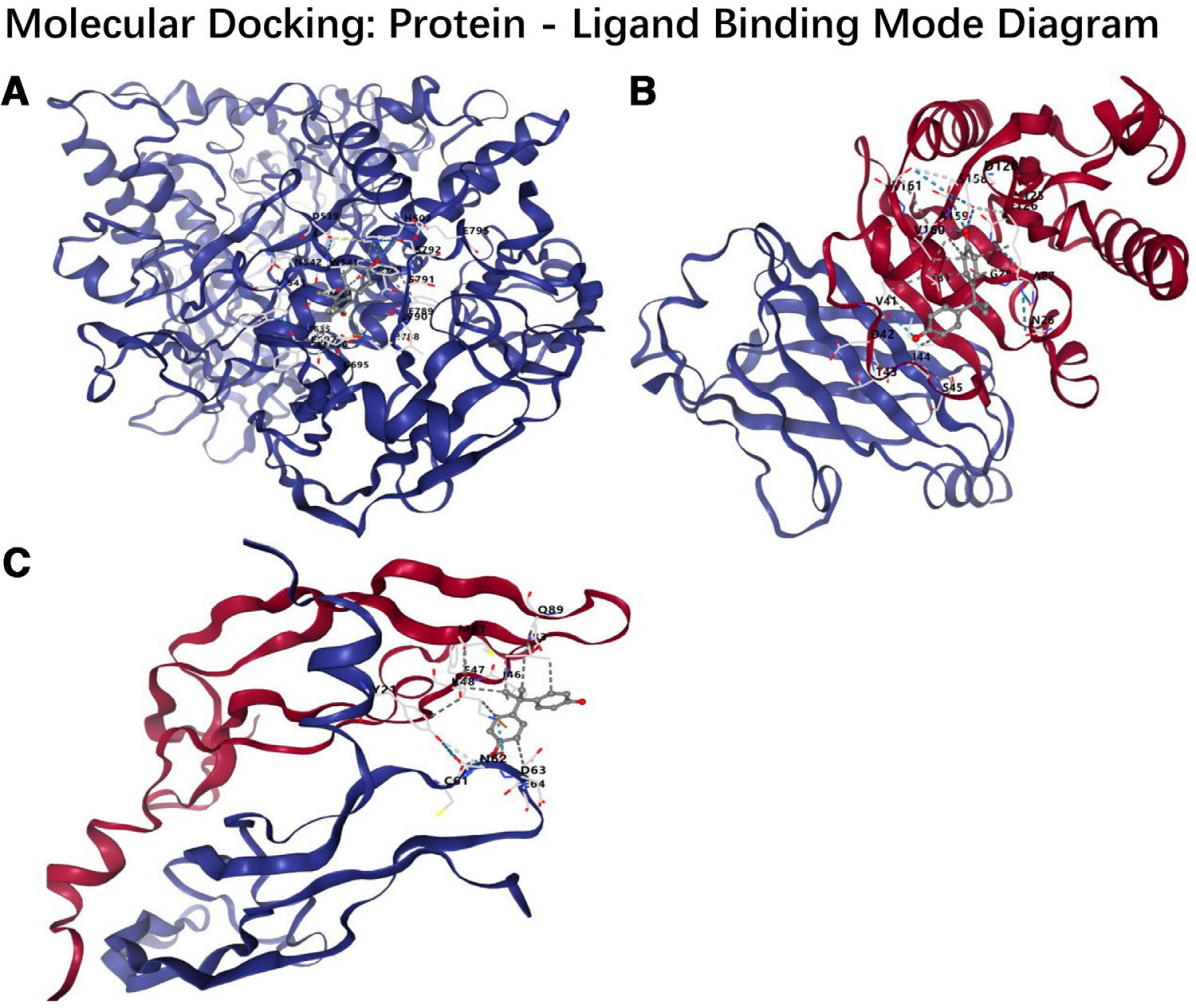
Protein–ligand binding mode diagram. (A) BPA–MANBA complex: lowest binding free energy = ‐8.1 kcal/mol; (B) BPA–PDE6D complex: lowest binding free energy = ‐6.7 kcal/mol; (C) BPA–VEGFA complex: lowest binding free energy = ‐6.1 kcal/mol. BPA = bisphenol A, VEGFA = vascular endothelial growth factor A.

The protein structure of MANBA was retrieved from the AlphaFold Database, while those of PDE6D and VEGFA were obtained from the RCSB PDB Database. The ligand structure of BPA was sourced from the PubChem Database. Molecular docking was performed using the CB-DOCK2 server. The lowest binding free energies of BPA with MANBA, PDE6D, and VEGFA were calculated as ‐8.1 kcal/mol, ‐6.7 kcal/mol, and ‐6.1 kcal/mol, respectively. The docking models are shown in Figure 7.

### 3.4. Molecular dynamics simulation

**Figure 8.**
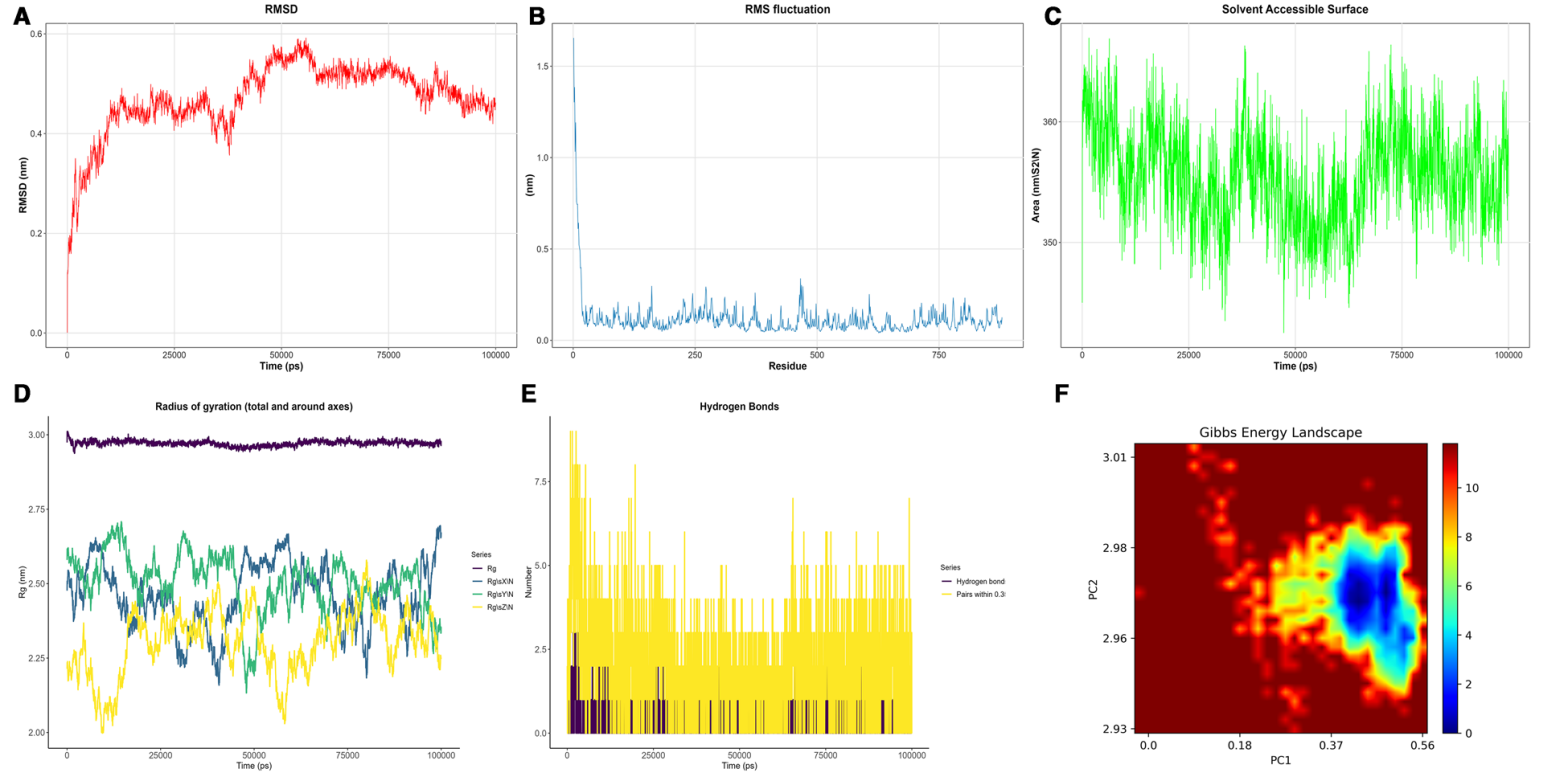
Molecular dynamics simulation of BPA–MANBA complex. (A) RMSD showed initial rise then stabilization, indicating system equilibrium; (B) RMSF indicated most residues were rigid except N-terminal (high fluctuation); (C) SASA remained stable, confirming no structural change; (D) hydrogen bonds were abundant between BPA and MANBA; (E) Rg stability reflected compact structure; (F) Gibbs energy landscape showed stable binding in low-energy (blue) regions. BPA = bisphenol A, Rg = radius of gyration, RMSD = root mean square deviation, RMSF = root mean square fluctuation; SASA = solvent accessible surface area.

The root mean square deviation was used to analyze the structural stability of the system. The curve initially rose and then stabilized with a slight decline, indicating that the system reached a relatively stable state over time (Fig. 8A). The root mean square fluctuation reflects the flexibility of individual residues (or atoms) during simulation. Most residues showed low fluctuation values, suggesting rigid and stable structures, while a few residues (especially N-terminal residues) exhibited high fluctuation and flexibility (Fig. 8B). The solvent accessible surface area monitors dynamic changes in the protein surface exposed to the solvent. In our study, the solvent accessible surface area of the protein system remained relatively stable, indicating no significant unfolding or contraction of the overall protein structure (Fig. 8C). The hydrogen bonds analysis tracked the number of hydrogen bonds between the protein and ligand, showing abundant binding sites in our model (Fig. 8D). The radius of gyration reflects the compactness of the protein system. The stable radius of gyration during simulation indicated high overall stability of the protein (Fig. 8E). The Gibbs energy landscape depicts free energy changes and dominant conformational clusters: the deeper blue regions represented lower free energy, indicating more stable binding between the ligand and protein (Fig. 8F).

## 4. Discussion

This study evaluated the toxicity of BPA and applied network toxicology to screen 92 genes associated with both BPA and SNHL. GO and KEGG enrichment analyses were carried out for the purpose of characterizing these genes. To further clarify the causal association between genes and SNHL, MR analysis was conducted, identifying one protective gene (MANBA) and 2 pathogenic genes (PDE6D and VEGFA). Notably, MR has emerged as a robust tool for disentangling causal relationships in hearing loss research, as demonstrated by its successful application in identifying inflammatory proteins (e.g., CCL19) as common risk factors for multiple hearing loss subtypes.^[20]^ Molecular docking showed that the binding free energies of these 3 genes with BPA were all less than ‐5 kcal/mol, indicating stable binding and suggesting that BPA may exert ototoxic effects through these genes. Molecular dynamics simulation further confirmed the dynamic stability of MANBA–BPA binding, providing a theoretical basis for investigating the molecular mechanisms of BPA-induced deafness. Collectively, these results are robust and improve our grasp of the underlying mechanisms.

At the regulatory level of the PI3K–Akt signaling axis, BPA exposure inhibits PI3K–Akt pathway activity through epigenetic modifications (such as DPY30-mediated H3K4me3 methylation), a mechanism that has been validated in testicular toxicity models.^[21]^ In the inner ear, the PI3K–Akt pathway serves as a critical protective mechanism for hair cell survival. Deletion of Akt1 significantly increases sensitivity to noise-induced hearing loss, while the pathway activator pasireotide protects hair cells by upregulating antiapoptotic proteins.^[22,23]^ Additionally, BPA may downregulate the expression of potassium channels (such as KCNQ4) in pancreatic islet β-cells via the ERβ receptor, a process associated with PI3K–Akt pathway-mediated transcriptional regulation.^[24]^ Recent studies have confirmed that KCNQ4 deficiency alone is sufficient to trigger widespread dysfunction in the Organ of Corti and induce progressive SNHL, including genetic and noise-induced subtypes.^[25]^ Collectively, BPA-mediated inhibition of the PI3K–Akt pathway may impact auditory function through 2 mechanisms: impairing hair cells’ resistance to oxidative stress and interfering with membrane trafficking and activity regulation of potassium channels (e.g., Kv1.3, Kv1.5), with KCNQ4 dysfunction being a key driver of cochlear ion homeostasis imbalance and outer hair cell (OHC) death, ultimately leading to cochlear ion homeostasis imbalance.^[25,26]^ Consistently, Tang et al^[16]^ demonstrated that BPA-induced ER-α activation in cochlear hair cells promotes mitochondrial reactive oxygen species overproduction, which not only disrupts mitochondrial membrane potential but also triggers pyroptosis (this finding directly validates our toxicology analysis showing BPA’s targeting of ER-α and mitochondrial function, and further identifies pyroptosis as a novel downstream pathway of BPA-induced ototoxicity).

MR analysis indicated that MANBA acts as a protective factor for SNHL, a finding highly consistent with the phenotypes of diseases associated with MANBA mutations. It has been found that a 13.1 kb inverted repeat mutation within the MANBA gene leads to loss of lysosomal β-mannosidase activity. Patients carrying this mutation exhibit SNHL accompanied by pathological features such as angiokeratoma.^[27]^ Another study on the Czech and Slovak Roma population identified a prevalent ethnic-specific MANBA splice-site variant (c.2158-2A>G); homozygous carriers of this variant consistently present with hereditary hearing loss, further confirming that MANBA dysfunction drives auditory impairment.^[28]^ Our molecular docking and molecular dynamics simulation results showed stable binding between BPA and MANBA. We infer that BPA may inhibit MANBA expression through epigenetic modifications (such as DNA methylation) or transcriptional interference, weakening its function in maintaining lysosomal homeostasis and thereby exacerbating metabolic damage to inner ear hair cells. The protective effect of MANBA likely originates from its regulation of lysosomal enzyme activity, while BPA can bind to it stably and suppress its expression.

In our study, PDE6D is a risk factor for SNHL. Previous studies^[29,30]^ have found that its functional disorder is directly associated with abnormal development of inner ear hair cells. Chessum et al confirmed that the transcription factor Ikzf2 (Helios) can specifically activate the expression of PDE6D in OHCs, and the abnormal up-regulation of this gene will interfere with the differentiation trajectory of inner hair cells into OHCs.^[29]^ Studies have shown that PDE6D, as a molecular marker for OHCs maturation, its expression imbalance leads to disorders of hair cell polarity differentiation, affecting auditory signal transduction.^[29,30]^ Considering the endocrine-disrupting properties of BPA, it may activate the response elements in the promoter region of PDE6D by mimicking estrogen and inducing ER-dependent epigenetic modifications (e.g., DNA methylation) (a mechanism supported by BPA’s regulatory effects on the methylome in hormone-sensitive cell).^[31]^ Additionally, as a member of the cAMP phosphodiesterase family, the abnormal expression of PDE6D may disrupt the dynamic balance of cAMP in inner ear cells, thereby affecting the gating properties of potassium ion channels (such as Kcne3) and the stability of the cytoskeleton.^[32,33]^

In the association between BPA and SNHL, VEGFA, identified as a harmful factor through MR analysis, may exert its effects by regulating inner ear vascular homeostasis and cellular signaling pathways. Studies have shown that BPA exposure can upregulate VEGFA expression: environmentally relevant doses of BPA (0.1 nM–1 μM) increase VEGF-A mRNA expression in human primary endothelial cells,^[34]^ and BPA also rapidly induces VEGF isoform expression in rat tissues.^[35]^ Other studies have shown that VEGFA is a core target of resveratrol in the treatment of sudden SNHL and participates in hair cell apoptosis through the PI3K–Akt pathway. Additionally, VEGFA can induce the expression of potassium channel genes (such as Kcne3), which are specifically expressed in vascular endothelial tip cells. Abnormalities in these genes may disrupt potassium ion homeostasis in the inner ear, leading to impairments in auditory signal transduction.^[32]^ These observations are consistent with our enrichment analysis showing that PI3K–AKT pathway abnormalities are closely linked to SNHL. Notably, studies on age-related hearing loss have shown that reduced VEGFA expression in the cochlea is associated with vascular abnormalities, which in turn exacerbates the occurrence of deafness.^[36]^ This contradicts our research findings, potentially due to differences in research models: reduced VEGFA expression in age-related hearing loss is associated with vascular degeneration (protective effect), whereas abnormal activation of VEGFA under BPA exposure may mediate ototoxicity through pro-inflammatory or pro-angiogenic mechanisms. The cellular microenvironment, VEGFA expression levels, and signaling pathways differ between the 2 scenarios. Molecular docking results show that VEGFA binds stably to BPA, further supporting that BPA may directly act on VEGFA protein to disrupt its normal regulatory network, ultimately driving the occurrence of SNHL.

Although this study provides a theoretical ototoxic mechanism linking BPA to SNHL, several limitations remain. A key limitation is the lack of clarification regarding the timing and multilevel mechanisms underlying BPA-induced SNHL, particularly the sequential interplay between upstream influences and downstream pathological processes. Specifically, factors such as BPA exposure and gene–environment interactions trigger molecular and signaling alterations (e.g. potassium channel dysfunction and MAPK/PI3K–Akt pathway dysregulation) upstream, which subsequently lead to cochlear cell damage and impaired hearing function downstream. In terms of timing, BPA-induced molecular alterations occur in the short term after exposure, while cumulative cellular damage and overt hearing impairment manifest in the medium to long term. Mechanistically, molecular signaling abnormalities serve as the critical link between upstream exposure and downstream cellular pathology. In the MR analysis, while weak IV bias was excluded, the ORs for the associations between MANBA, PDE6D, VEGFA, and SNHL were close to 1, indicating a low effect size. And we did not perform multiple testing correction such as FDR or Bonferroni adjustment for the *P*-values of these associations. This may elevate the risk of false-positive findings, particularly since the MR analysis initially included a large number of genes, 41 in total, and thus requires cautious interpretation of the observed associations. Large-scale eQTL analyses have highlighted that polygenic effects and trans-regulatory mechanisms contribute substantially to gene expression variations associated with complex traits, which may partially explain the modest effect sizes observed in our MR results.^[37]^ This may be constrained by the ethnic homogeneity of the European population cohort, as well as the failure to stratify by age, gender, and phenotypic heterogeneity of hearing loss (e.g., sudden deafness, presbycusis, noise-induced hearing loss). Additionally, pleiotropy of genetic variants may confound causal inference. Although molecular docking and MANBA dynamic simulation confirmed the binding stability of BPA, they only reflected single protein–ligand interactions without modeling the coordinated regulation of AKT/MAPK pathways and potassium channels in the inner ear hair cell microenvironment. Furthermore, the lack of cell or animal model experiments hinders validation of our conclusions.

## 5. Conclusion

This study employs network toxicology, MR, molecular docking, and molecular dynamics simulation to demonstrate that BPA exerts ototoxic effects on SNHL through potassium channel-related processes and signaling pathways such as MAPK and PI3K−Akt. Three genes (MANBA, PDE6D, and VEGFA) show causal associations with the disease, and their protein structures stably bind to BPA ligands, suggesting that BPA may participate in the pathogenesis of SNHL by regulating these genes. Future research will expand sample sizes, add subgroup analyses, and perform functional experiments in cell and animal models to provide additional evidence for these conclusions.

## Acknowledgments

We would like to acknowledge the participants and investigators of the FinnGen consortium, eQTLGen consortium. We also thank the following public databases for contributing essential data: ProTox, PubChem, OMIM, GeneCards, Chembl, STITCH, SWISS, eQTLGen, FinnGen, RCSB PDB, AlphaFold, CB-DOCK2, and KEGG. Their resources were integral to the toxicological analysis, gene screening, and molecular modeling conducted in this study.

## Author contributions

**Data curation:** Xin Yan, Junmei Xuan, Jianghua Peng.

**Formal analysis:** Xin Yan, Jianghua Peng.

**Funding acquisition:** Junmei Xuan, Jianghua Peng.

**Investigation:** Xin Yan, Jianghua Peng.

**Methodology:** Xin Yan, Junmei Xuan.

**Writing – original draft:** Xin Yan.

**Writing – review & editing:** Jianghua Peng.
